# Hijacking Cellular Stress Responses to Promote Lifespan

**DOI:** 10.3389/fragi.2022.860404

**Published:** 2022-03-24

**Authors:** Naibedya Dutta, Gilberto Garcia, Ryo Higuchi-Sanabria

**Affiliations:** Leonard Davis School of Gerontology, University of Southern California, University Park Campus, Los Angeles, CA, United States

**Keywords:** mitochondria, endoplasmic reticulum, heat-shock, stress, aging, *C. elegans*

## Abstract

Organisms are constantly exposed to stress both from the external environment and internally within the cell. To maintain cellular homeostasis under different environmental and physiological conditions, cell have adapted various stress response signaling pathways, such as the heat shock response (HSR), unfolded protein responses of the mitochondria (UPR^MT^), and the unfolded protein response of the endoplasmic reticulum (UPR^ER^). As cells grow older, all cellular stress responses have been shown to deteriorate, which is a major cause for the physiological consequences of aging and the development of numerous age-associated diseases. In contrast, elevated stress responses are often associated with lifespan extension and amelioration of degenerative diseases in different model organisms, including *C. elegans*. Activating cellular stress response pathways could be considered as an effective intervention to alleviate the burden of aging by restoring function of essential damage-clearing machinery, including the ubiquitin-proteosome system, chaperones, and autophagy. Here, we provide an overview of newly emerging concepts of these stress response pathways in healthy aging and longevity with a focus on the model organism, *C. elegans.*

## Introduction

Rejuvenation of human life by reducing the damaging effects of aging is one of the trending focuses of current biological research across the globe. Aging is an obvious physiological condition for all living organisms on this planet. It has been designated as a cumulative impairment of different cellular events, which are mainly associated with the maintenance of cellular homeostasis. This frailty condition, broadly influenced by various stressors or genetic factors, leads to the development of major disease conditions including diabetes, neurodegenerative disorders, cardiovascular disorders, and cancer, all of which together increases the vulnerability to death (Franceschi et al., 2018). In 2014, Carlos López-Otín et al. has characterized nine major factors as the hallmark of aging: genomic instability, telomere attrition, epigenetic alterations, and loss of protein homeostasis (proteostasis) categorized as the primary hallmarks of aging; stem cell exhaustion and altered intercellular communication referred to as integrative hallmarks; and–mitochondrial dysfunction, deregulated nutrient sensing, and cellular senescence categorized as antagonistic hallmarks (López-Otín et al., 2013).

Stress, generated from different endogenous or exogenous sources can disturb homeostasis across cellular compartments, contribute to cellular dysfunction, and impact the aging process (Li et al., 2021). As such, living organisms have acquired different unique cellular signaling pathways through evolution to cope with the constant exposure of different internal or external stresses (Kirkwood and Austad 2000). These responses generally include the activation of a widespread transcriptional program to promote genes important for restoring cellular homeostasis. These include the heat-shock response (HSR) and organelle-specific stress response pathways like the endoplasmic reticulum unfolded protein response (UPR^ER^) and the mitochondrial unfolded protein response (UPR^MT^), which have been studied in different organisms, including *Caenorhabditis elegans* (Kenyon 2010; Kourtis and Tavernarakis 2011). Often, it is the breakdown or dysregulation of these important cellular quality control and stress response machineries that are causative of aging. For example, in neurodegenerative disorders like Parkinson’s disease (PD), Alzheimer’s disease (AD), and Huntington’s disease (HD), misfolded protein accumulation caused by compromised activation of stress responses lead to decreased health. In addition, dysfunction of different organelles such as the mitochondria and endoplasmic reticulum under these conditions can also facilitate neurodegenerative diseases and metabolic disorders (Ozcan and Tabas 2012; Hartl 2017; Colla 2019; Misrani et al., 2021).

Using *C. elegans* as a model system, research over the last few decades has established the importance of the activation of cellular stress response pathways in healthy life. Stimulation of stress response pathways can restore cellular homeostasis and reduce the risk of the manifestation of age-related diseases, so lengthening life span. In this context, short exposure to mild stress can be beneficial to organismal health and longevity. Termed hormesis, early exposure to stress can activate critical cellular stress responses that can mitigate the accumulation of damage at advanced age (Rattan 2004). Many of these pathways, which include the HSR, UPR^MT^, and UPR^ER^ have originally been identified in *C. elegans*, again highlighting the strength of this model in stress biology. In this review, we sum up our latest understanding on the significance of successful stress response pathways in longevity, distinctly emphasizing the model organism *C. elegans*.

### The Cytosolic Heat-Shock Response

In 1962 an accidental finding by an Italian scientist Ferruccio Ritossa opens a new window in biological research. He observed a different and unique puffing pattern in the polytene chromosome of the salivary cells of the fruit fly, *Drosophila* busckii under an elevated temperature condition (Ritossa 1962; Ritossa 1996). That unknown chromosomal puff was later identified as the active transcriptional region for the synthesis of a special group of proteins known as heat shock proteins, which maintain proteostasis in a cell by facilitating protein stabilization, refolding of misfolded protein structure, protein translocation, and degradation of toxic protein aggregates (Tissiéres et al., 1974; Lindquist and Craig 1988; Anckar and Sistonen 2011). These heat shock proteins are conserved across living organisms and considered as the principal functional unit for the HSR. Heat shock proteins are widely grouped into two families based on their molecular weight: First, the large ATP-dependent heat shock proteins of molecular mass between 40 and 105 kDa, including the major chaperone proteins, HSP70, HSP90, etc., and second, the small heat shock proteins of molecular mass between 8 and 25 kDa, which includes HSP27, HSP25, ubiquitin, and others (Jee 2016).

Most protein chaperones share similarities in their function. However, they differ in respect to their cellular localization, substrate specificity, and mechanistic details. The HSP70 family make up the most abundant chaperone proteins present in the cell. The cytosolic HSP70 has several homologs, which are present in different subcellular compartments including the heat shock cognate 70 (HSC70) present in the cytosol along with HSP70 and GRP78/BiP present in the endoplasmic reticulum (Radons 2016). HSP90 is another ATP-dependent chaperone protein that is constitutively expressed in cells and found in different cellular compartments like the cytosol, mitochondria, endoplasmic reticulum, nucleus etc. HSP90 shows the specificity to bind with a large number of misfolded proteins and provide support to refold to their functional state (Schopf et al., 2017). In contrast to the ATP-dependent chaperone proteins, small heat shock proteins function in an ATP-independent manner. These small heat shock proteins such as HSP27 and αβ-crystallin show induced expression in response to stress. During proteotoxic stress, they bind with misfolded client proteins and block further misfolding and/or the formation of misfolded protein aggregates until the clients are delivered to repairing chaperones like HSP70 and HSP40 (Bakthisaran et al., 18542015; Schopf et al., 2017). In addition to protein refolding, many molecular chaperones also participate in proteasomal degradation. For example, chaperone molecules can couple with ubiquitin ligase proteins that facilitate the polyubiquitination of misfolded proteins and prepare them for proteasomal degradation.

### HSF1 and the Heat Shock Response

Heat shock proteins are synthesized in a large amount immediately after sensing stress, such as elevated temperature, hypoxia, exposure to heavy metals, etc. This universally conserved cellular stress response is orchestrated by a key transcription factor known as heat shock factor 1 or simply HSF1 (Anckar and Sistonen 2011). In invertebrates, the heat shock factor is encoded by a single gene, whereas in vertebrates, four major heat shock factor isoforms, HSF1, HSF2, HSF3, and HSF4 have evolved. In the case of both vertebrates and invertebrates, HSF1 plays the major decisive role in the activation of the HSR while the other isoforms that exist only in vertebrates are less studied and found to show some unique and sometimes tissue-specific functions. Interestingly, while HSF1, HSF2, and HSF4 have been found in all vertebrates, HSF3 was specifically observed in avian species only (Nakai et al., 1995). HSF2 plays an important role in female fertility, spermatogenesis, and early development, whereas HSF4 participates in eye lens development (Schuetz et al., 1991; Nakai et al., 1997).

**FIGURE 1:**
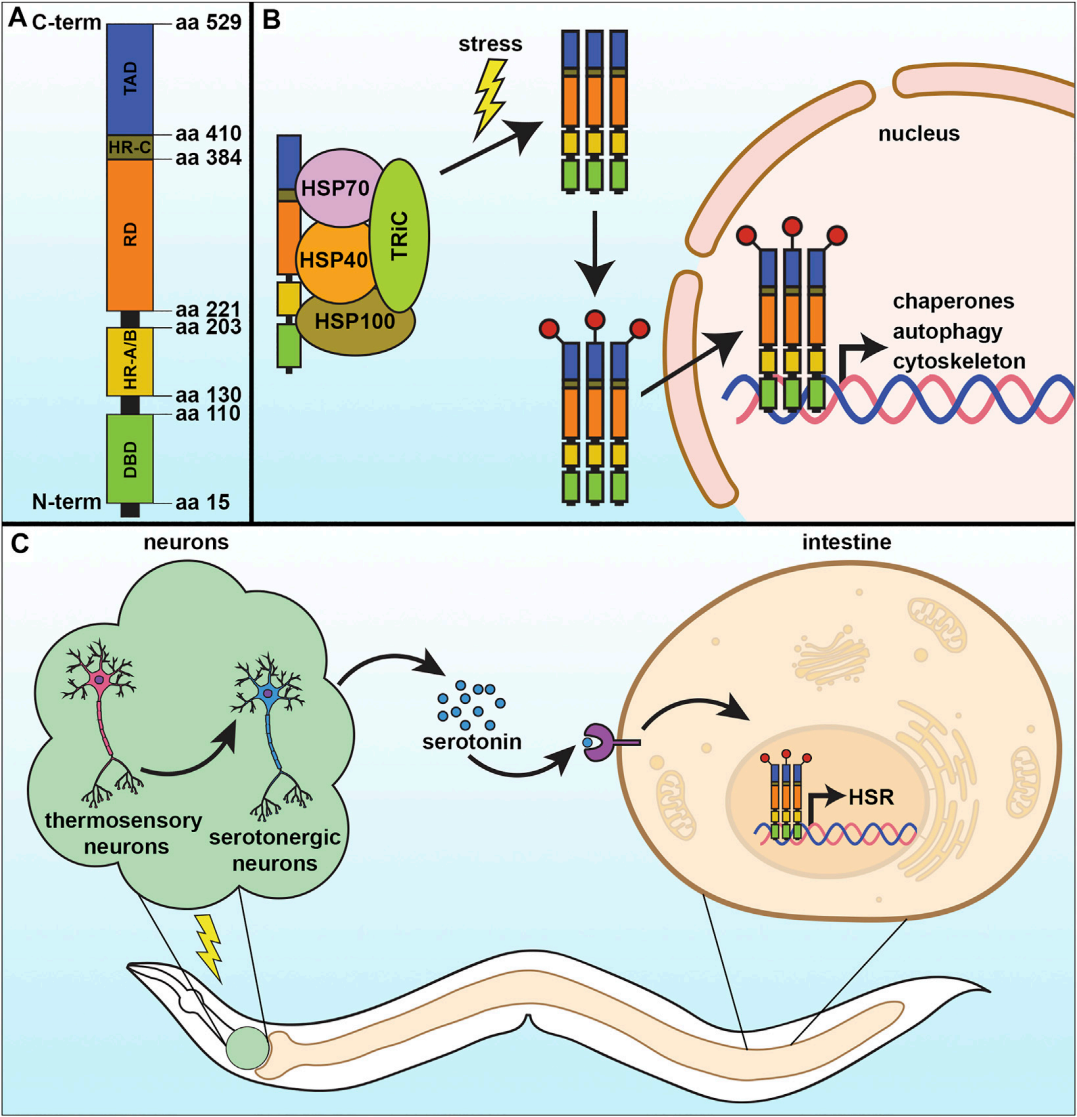
The cytosolic heat-shock response. **(A)** The heat-shock response (HSR) is regulated by the transcription factor, HSF-1, which contains four major structural domains: the DNA-binding domain (DBD), regulatory domain (RD), trans-activating domain (TAD), and the oligomerization domain divided into HR-A and HR-B. **(B)** Under basal conditions, HSF-1 is bound by regulatory proteins that prevent its activation. Upon stress, these proteins are released from HSF-1 as they are titrated away to serve as molecular chaperones to damaged proteins, allowing HSF-1 to trimerize and serve as a transcription factor to activate genes important to mitigate damage and increase survival. **(C)** The HSR can be communicated in a non-autonomous manner, whereby thermosensory and serotonergic neurons can communicate to peripheral tissue, including the intestine, to activate a systemic HSR and promote organismal health and lifespan.

HSF1 is made of 529 amino acids and its structure consists of four major structural/functional domains: the DNA binding domain (DBD), oligomerization domain, regulatory domain (RD), and transactivation domain (TAD) (Figure 1A). The DBD is present at the amino-terminal end of HSF1 and forms a helix turn helix structure. Through this domain, HSF1 binds with its specific DNA sequence and that association is stabilized by the interaction between the amphipathic helical region and the hydrophobic DNA pocket (Neudegger et al., 2016). Next to the DBD, the alpha-helix rich oligomerization domain is situated through which one HSF1 molecule interacts with another (Peteranderl et al., 1999). That oligomerization domain is further divided into two subdomains HR-A and HR-B. A separate and similar domain HR-C is present between the transactivation domain and the regulatory domain that helps to inhibit random oligomerization of HSF1 (Anckar and Sistonen 2011; Rabindran et al., 1993). The regulatory domain, present alongside the oligomerization domain (between HR-A/HR-B and HR-C) controls the transactivation of HSF1 and is the site for regulatory post-translational modifications, such as phosphorylation, acetylation/deacetylation, and sumoylation (Anckar and Sistonen 2011). Ultimately at the C-terminus of the HSF1 protein is the TAD, which facilitates the transcriptional activation of its target genes. However, this is the most structurally unknown part of the protein to date. In normal growth conditions, monomeric HSF1 exists as an inactive form in the cytoplasm in association with a complex of some regulatory proteins such as HSP70, HSP90, and TRiC. These molecular chaperones act as a negative regulator for HSF1 function (Anckar and Sistonen 2011; Shi et al., 1998; Neef et al., 2014). In response to stress, the monomeric HSF1 is released from its inhibitory complex and trimerizes, allowing for a series of post-translational modifications which help in its nuclear translocation and conversion to an active DNA binding component (Figure 1B). Those post-translational modifications include phosphorylation, sumoylation, acetylation, and deacetylation, which are vital to the activation-attenuation cycle of HSF1 (Anckar and Sistonen 2011; Dai 2018). HSF1 binds to a conserved pentameric sequence motif termed the heat shock element or HSE (5′nGAAn3′) at the promoter of its target genes, which include the heat shock proteins, autophagy, actin, and innate immunity (Anckar and Sistonen 2011).

### Heat Shock Response and Aging

As a critical player in cellular stress response, HSF1 function can have a direct impact on physiological health. However, like most quality control mechanisms, HSF1 activity, and the HSR deteriorate during the aging process. Specifically, the HSR in *C. elegans* exhibits decreased transcriptional output early in adulthood (Labbadia and Morimoto 2015). Shockingly, there is a precipitous drop in the induction of HSR genes (including *hsp-70* and *hsp-16.11*) in response to thermal stress within a narrow 4-h window that coincides with the start of egg-laying. Interestingly, this collapse of the HSR was not due to the decreased binding ability of HSF-1, but rather due to profound changes to the chromatin landscape due to the overt decline in expression of the histone demethylase *jmjd-3.1*. This decreased functional output of the HSR has direct physiological ramifications, as animals exhibit an age-dependent decrease in thermotolerance and an increase in protein aggregation load (Morley et al., 2002; Ben-Zvi et al., 2009). The repression of HSR can be compensated by increased expression of *hsf-1* to diminish the burden of proteotoxicity at advanced age to improve lifespan (Morley and Morimoto 2004; Ben-Zvi et al., 2009; Shemesh et al., 2013). Specifically, overexpression of *hsf-1* can significantly extend lifespan (Hsu et al., 2003; Baird et al., 2014), whereas downregulation shortens lifespan and results in premature aging phenotypes (Garigan et al., 2002). Knockdown of the downstream targets of HSF-1, including the heat shock proteins, HSP70 and HSP90 also decreases lifespan (Morley and Morimoto 2004), whereas long-lived mutants including *daf-2* and *age-1* show increased expression of these chaperones (Hsu et al., 2003; McElwee et al., 2003; Murphy et al., 2003), providing further evidence that HSF-1-mediated stress resilience is important for longevity. In fact, longevity can actually be predicted by the expression of a single target of HSF-1, *hsp-16.2* (Mendenhall et al., 2012). Importantly, HSF-1 has a beneficial effect on multiple tissue types, as overexpression solely in neurons, the body wall muscle, or intestinal cells was sufficient to promote lifespan extension (Morley and Morimoto 2004). Finally, *hsf-1* has a temporal requirement in lifespan extension, whereby its expression during development impacts lifespan, whereas overexpression later in adulthood has no effect (Volovik et al., 2012).

Beyond just an ectopic expression of *hsf-1*, activation of the HSR through exposure to low levels of thermal stress have also been shown to impact longevity (Butov et al., 2001). Termed hormesis, this described the phenomenon whereby low-grade exposure to stress can be beneficial to organismal health by activating critical stress response pathways. While these beneficial effects of hormesis were primarily ascribed to heatshock proteins and DAF-16 (insulin growth factor) signaling (Cypser and Johnson 2002; Hsu et al., 2003; Morley and Morimoto 2004; Rattan 2005), it is becoming increasingly clear that heatshock proteins are not the only effector molecules. Based on several genome-scale studies, it has been recognized that HSF1 has a much broader functional spectrum that can control numerous molecular events associated with cellular quality control pathways, including ubiquitin-mediated proteasomal degradation, autophagy, and the maintenance of organelles including mitochondria, ER, and the actin cytoskeleton (Barna et al., 2018). In fact, hypomorphic mutations of *hsf-1* and perturbations of some HSR chaperones had no impact on thermotolerance (McColl et al., 2010; Kourtis et al., 2012). One study even found that overexpression of *hsf-1* with a truncation in the C-terminal transactivating domain dramatically extended lifespan, despite having no impact on the induction of heat-shock proteins (Baird et al., 2014). Instead, this variant of HSF-1 increased the expression of *pat-10*, a troponin-like calcium-binding protein that promoted actin cytoskeletal maintenance and function. This increase in cytoskeletal integrity at advanced age directly impacted organismal health and promoted longevity. Similar to the induction of heat-shock proteins (Morley and Morimoto 2004), HSF-1’s beneficial impact on the cytoskeleton independently affected multiple cell types to promote lifespan (Higuchi-Sanabria et al., 2018a).

HSF-1 has also been shown to function both alongside and as a regulator of autophagy. It can bind directly to the promoter of the autophagy component, ATG7, and induce its expression, which is independent of the canonical function of HSF1 (Desai et al., 2013). Furthermore, it has been also reported that casein kinase 1 phosphorylates the essential autophagy receptor SQSTM1/p62 to lift up the selective autophagic clearance in an HSF1 dependent manner (Watanabe et al., 2017). Induction and activation of the SQSTM1/p62 receptor is sufficient to induce autophagy in distinct tissues and for longevity (Kumsta et al., 2019). A recent study in *C. elegans* has explored the induction of HSF-1 by hormetic heat stress exposure, which can clear the accumulation of PolyQ aggregates and contributes to enhanced stress resistance and extended lifespan (Kumsta et al., 2017). Similarly, overexpression of *hsf-1* is sufficient to induce autophagy and induction of autophagy is required for the beneficial effects of HSF-1. However, a conflicting study found that HSF-1 can actually prevent autophagy by promoting HSP70 expression, which can inhibit starvation and rapamycin-induced autophagy (Dokladny et al., 2013). These seemingly contradicting results may be due to differences in thermal stress conditions. For example, it is entirely possible that under conditions of acute stress, HSF-1 can coordinate heat-shock proteins and chaperones to mitigate damage by protein remodeling. In contrast, under chronic stress when damage exceeds the repair capacity of chaperones, autophagy is essential. Regardless, it is clear that a proper balance between HSR and autophagy is essential for proper maintenance of homeostasis. Indeed, HSR and autophagy can be delicately balanced and coordinated under specific environmental stress and metabolic cues via the homeodomain interacting protein kinase, HPK-1 (Das et al., 2017). Specifically, HPK-1 sits at the center of a dual proteostatic network whereby it is essential for the induction of autophagy gene expression in response to dietary restriction and inactivation of mTOR, but also inhibits sumoylation of HSF-1 to promote its transcriptional activity upon thermal stress.

Studies about the cross-talk between the proteostatic network and immune response of an organism revealed that long-lived *C. elegans* mutants showed higher resistance to bacterial pathogens along with increased longevity (Garsin et al., 2003). In fact, one of the primary causes of death in C. elelgans is due to pathogenic invasion (Zhao et al., 2017), suggesting that factors that there may be heavy overlap between factors that increase lifespan and immunity. Thus, it is not surprising that HSF-1 has also been implicated in immune response. Specifically in *C. elegans*, HSP90 and the small heat shock proteins are important for the development of immunity. Interestingly, this HSF-1-mediated defense response did not require p38 MAPK, but recruits the DAF16 pathway for the development of multi-pathogen resistance (Singh and Aballay 2006). Similar to autophagy, this beneficial effect of the HSR is not limited to *hsf-1* overexpression and hormetic heat-shock can stimulate the immune response to promote resistance to pathogen exposure (Prithika et al., 2016). In higher eukaryotes with more complex immune systems, the function of induced heat shock proteins is not only limited to ameliorating inflammatory damage, but also encourage the production of anti-inflammatory cytokines (van Eden et al., 2005). HSF1 has been found as a transcriptional activator for the expression of interleukin 10 (IL10) which inhibits the bacterial lipopolysaccharide-mediated production of pro-inflammatory cytokines like TNFα, IL-12, IL-1b, IFNg (Zhang et al., 2012). Furthermore, the inflammatory stress-dependent and the HSF1 mediated induction of heat shock proteins plays an essential role in preparing peptides for proper antigen presentation by CD8^+^ T lymphocytes (Binder and Srivastava 2005).

### Neuronal Transmission of the Heat Shock Response

In metazoans, the HSR can also be activated non-autonomously irrespective of the presence of thermal stress. It has been reported in rats for the first time by Fawcett et al. that HSF1 activation can be governed by the nervous system in the absence of environmental stress. That study revealed that controlled stress conditions release adrenocorticotropin from the pituitary gland of Wistar rats that facilitate HSF1 trimerization and increase its DNA binding activity, which consequently induces HSP70 expression in adrenal tissue (Fawcett et al., 1994). Later, a similar mechanism involving the nervous system in proteostatic regulation has been observed in *C. elegans*, where the HSR of somatic cells was found to be controlled in a cell non-autonomous manner by two thermosensory AFD neurons, GCY-3, and TTX-3 (Figure 1C) (Prahlad et al., 2008a). Optogenetic stimulation of the AFD neurons communicates with two serotonergic neurons ADF and NSM that releases serotonin, which was sufficient to activate HSF1 in another cell. In the receiving cell, the serotonin receptor SER-1 drove increased synthesis of chaperone proteins. They firmly established that activation of serotonergic neurons is sufficient to reduce a load of misfolded protein accumulation in *C. elegans* (Tatum et al., 2015). To survive in nature an organism needs to have the ability to react rapidly to environmental challenges. Thus, it is not surprising that organisms with a developed nervous system have neurons which can sense stress and relay a signal to other tissue. Indeed, neurons can also sense an olfactory experience to prime the function of HSF1 to provide a rapid and stronger response to subsequent exposure to any proteotoxic agents (Ooi and Prahlad 2017). Stimulation of the olfactory neurons by the odorants produced by the toxic bacterium *P. aeruginosa* can enhance the pathogen-avoiding ability in *C. elegans* in an HSF-1 and serotonin-signaling dependent manner.

Importantly, one study found that overexpression of *hsf-1* in neurons was sufficient to drive increased HSR and DAF-16/FOXO activity in peripheral tissue (Douglas et al., 2015). This combined activation of HSF-1 and DAF-16 targets was sufficient to increase thermotolerance and lifespan, which was similar to the increase in lifespan found in animals with reduced insulin signaling (Son et al., 2018). In addition, neuronal *hsf-1* was sufficient to drive protection of the actin cytoskeleton in multiple cell types during aging, including the muscle, hypodermis, and intestine, which was equally important for the increased longevity of these animals. This is curious considering the previously identified involvement of integrins in actin function (Tharp et al., 2021) and HSF-1-mediated depletion of integrin-linked kinase in increased stress resistance and longevity in *C. elegans* (Kumsta et al., 2014). These data beg the question of whether non-autonomous HSF-1 signaling could potentially utilize similar integrin signaling mechanisms to drive longevity. Finally, neuronal *hsf-1* signaling also directly impacts fat metabolism and results in extensive fat remodeling. Specifically, neuronal *hsf-1* results in decreased expression of the fat desaturases *fat-6/fat-7*, while activating the expression of catabolic lysosomal lipases, which shifts the fatty acid composition of the plasma membrane to a more saturated state. This change in fat mimics those downstream of exposure to thermal stress, and is sufficient to drive long-term survival of animals at elevated temperature (Chauve et al., 2021).

Beyond nonautonomous signaling from neurons to periphery, a recent study has found that serotonin can actually transmit HSF-1 signaling to future progeny (Das et al., 2020). Serotonin, released from the maternal neurons can ensure higher longevity and stress resilience in their future offspring through HSF-1 activation in germ cells. Specifically, serotonin signaling promotes protein kinase A (PKA)-dependent modification of HSF-1, increasing the occupancy of RNA Pol II and HSF1 at the promoter of various protective genes, including molecular chaperones which are the targets of HSF1 in response to even minimum heat stress. In addition, HSF-1 promotes the recruitment of a chromatin remodeler FACT (Facilitates Chromatin Transcription) to alter histone dynamics to initiate transcription. This mechanistic pathway is also conserved in mammalian cells. Overall, these studies highlight the numerous pathways HSF-1 modulates to impact organismal health and lifespan.

### Perspectives and Concluding Remarks

Beyond its clear role in regulating the HSR, HSF-1 has now been ascribed to several other equally important processes, including autophagy, immune response, and maintenance of the cytoskeleton. Considering its numerous functions, one intriguing question is how this single transcription factor can coordinate such diverse processes, and whether exposure to specific types of stressors can titrate HSF-1 to promoters of appropriate genes. For example, upon deletion of its C-terminal domain, HSF-1 can no longer induce heat-shock proteins, but strongly upregulates genes involved in cytoskeletal maintenance (Baird et al., 2014). It is possible that the C-terminal domain contains an important residue that allows for coordination with other transcriptional regulators recruits HSF-1 to heat-shock proteins, and loss of this domain causes increased accumulation of HSF-1 to cytosolic targets. Still to be understood is the identity of these potential cofactors that would allow HSF-1 to be targeted to specific gene loci.

Another intriguing question is how all the functional roles of HSF-1 are coordinated. To date, separate studies have shown that the induction of chaperones (Morley and Morimoto 2004), cytoskeletal function (Higuchi-Sanabria et al., 2018b), and autophagy (Kumsta et al., 2017) are all necessary for the beneficial effects of HSF-1 activation, such that perturbing any individually can completely abrogate HSF-1-mediated longevity. And while studies with the C-terminal deletion of HSF-1 showed that cytoskeletal regulation can be separated from chaperone induction (Baird et al., 2014), whether the beneficial effects of this HSF-1 variant requires autophagy has yet to be identified. Importantly, all these processes have been shown to be induced upon exposure to heat-shock, suggesting that the majority of HSF-1 targets are simultaneously induced, rather than being separable. Thus, are these seemingly divergent responses actually distinct mechanisms, or all parts of the same pathway? Indeed, all of these pathways do converge into a similar goal: increased proteostasis through protein folding by chaperones, clearance of damaged proteins through autophagy, and even the actin cytoskeleton has clear implications in protein homeostasis (Gross and Kinzy 2007).

### The Mitochondrial Unfolded Protein Response

Mitochondrial fitness and function are inarguably significant to cell viability, due to their numerous functions, including energy production, regulating apoptotic and necrotic cell death, storing calcium and amino acids, lipid oxidation, and heat production. Mitochondrial dysfunction is linked to aging and several age-related diseases, including Alzheimer’s disease, Parkinson’s, and metabolic syndrome (Higuchi-Sanabria et al., 2018c; Moehle et al., 2019). This means that proper functioning of mitochondria is essential for a healthy physiological state. However, almost counterintuitively, a growing number of studies have shown that perturbations to mitochondrial function can actually lead to lifespan extension (Dillin et al., 2002; Liu et al., 2005; Owusu-Ansah et al., 2013; Yee et al., 2014). The primary reason for these seemingly contradictory observations is that impairment of mitochondria triggers the unfolded protein response of the mitochondria (UPR^MT^), which results in beneficial effects on organismal health (Durieux et al., 2011).

**FIGURE 2:**
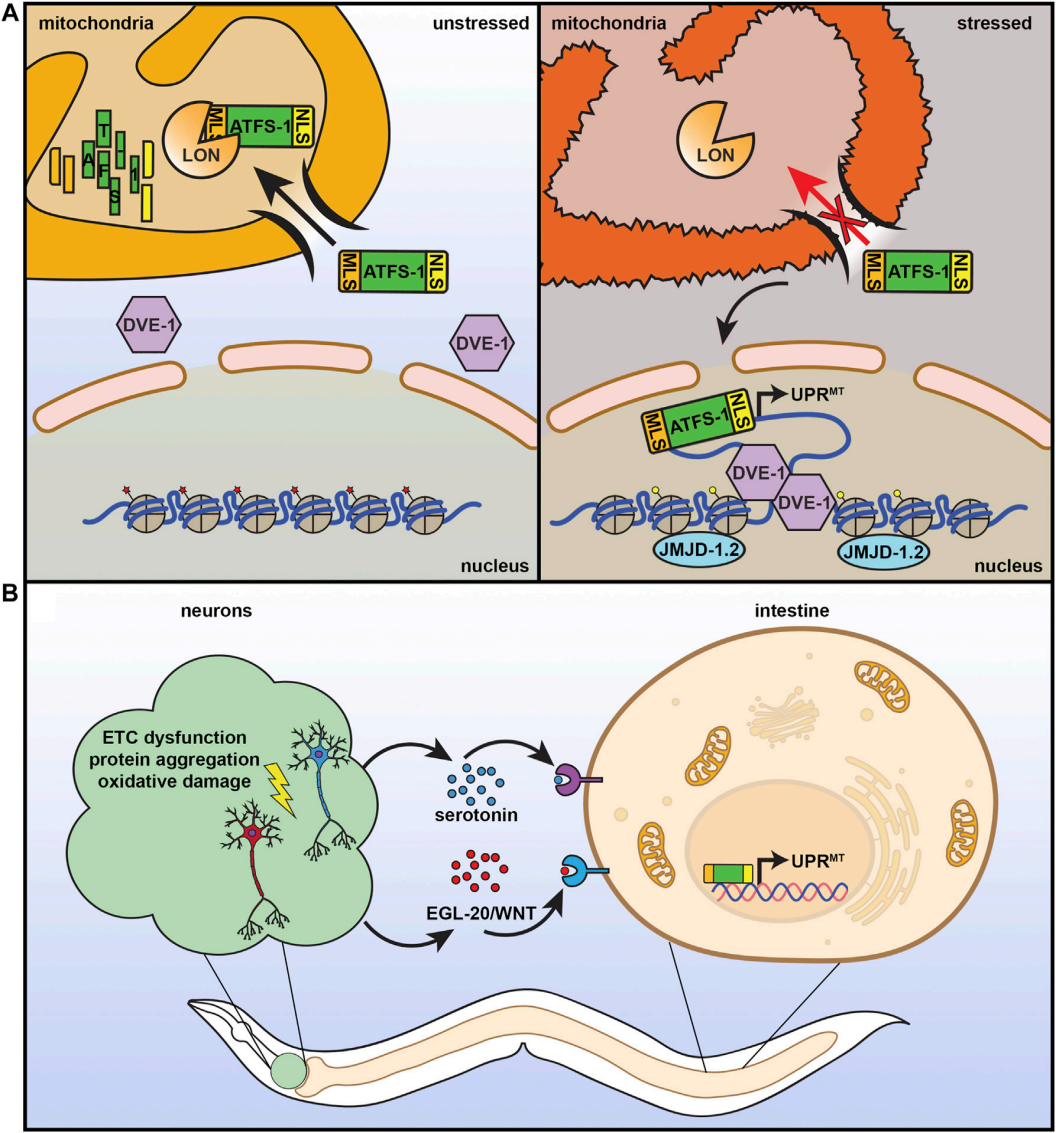
The mitochondrial unfolded protein response. **(A)** ATFS-1 is a unique protein that can serve as a sensor for mitochondrial health and fitness. It contains both a nuclear localization signal (NLS) and a mitochondrial localization signal (MLS). Under basal, unstressed conditions, ATFS-1 is imported into the mitochondria where it is degraded by LON protease. Under conditions of mitochondrial stress or damage, mitochondrial import decreases, allowing ATFS-1 to instead accumulate in the mitochondria where it can activate UPR^MT^ with additional transcriptional regulators including DVE-1 and chromatin regulator JMJD-1.2. **(B)** Similar to the HSR, UPR^MT^ can also be communicated in a nonautonomous manner. Neurons that experience mitochondrial stress can signal to the peripheral tissue, including the intestine, through serotonin and WNT signaling to result in systemic activation of UPR^MT^, increased stress resilience, and increased lifespan.

Considering the importance of mitochondria to cellular health, it is not surprising that cells have adapted many quality control mechanisms to protect mitochondrial function, including the UPR^MT^. UPR^MT^ is robustly activated by a variety of sources of mitochondrial stress: stoichiometric imbalance between proteins coded in the nuclear or mitochondrial genome (Couvillion et al., 2016; Houtkooper et al., 2013; Molenaars et al., 2020), impaired electron transport chain (ETC) function (Khalimonchuk et al., 2007; Zara et al., 2007; He et al., 2018), mitochondrial protein aggregation (Hallberg et al., 1993; Dubaquié et al., 1998), defects in mitochondrial import (Rolland et al., 2019; Xin et al., 2020), loss of mitochondrial membrane potential (Tharp et al., 2021), or disrupting mitochondrial translation (Nolden et al., 2005) and DNA replication (Kenny and Germain 2017). This process is best understood in *C. elegans*, where UPR^MT^ is regulated by several transcriptional regulators (Figure 2A). One specific transcription factor, ATFS-1, serves as a signal between the mitochondria and the nucleus. ATFS-1 contains both a nuclear localization sequence and a mitochondrial signal sequence and is preferentially imported into the mitochondria where it is subsequently degraded by mitochondrial Lon proteases. When mitochondrial import is compromised, such as under conditions of stress, ATFS-1 import is reduced and can accumulate in the nucleus to activate UPR^MT^ (Nargund et al., 2012). Several other transcriptional regulators work either in concert with, or independent of, ATFS-1 to regulate UPR^MT^, including the transcription factor DVE-1 and its ubiquitin-like cofactor UBL-5 (Benedetti et al., 2006; Haynes et al., 2007). Nuclear localization and transcriptional activation by DVE-1 is further promoted by the histone methyltransferase MET-2 and its cofactor LIN-65, which also induce epigenetic changes required for transmission of mitochondrial stress signaling between cells and through generations (Tian et al., 2016). Finally, histone demethylases JMJD-1.2 and JMJD-3.1 remodel chromatin to facilitate access to promoters of numerous UPR^MT^ target genes (Merkwirth et al., 2016).

At the genetic level, UPR^MT^ is characterized by a coordinated activation of genes essential for restoring mitochondrial fitness and function. Mitochondrial chaperones, such as Hsp70 and Hsp60/10 can refold misfolded proteins (Castro et al., 2018) and proteases, such as LONP and ClpP can degrade misfolded proteins (Wang et al., 1993) to alleviate proteotoxic stress. Importantly, proteins involved in mitochondrial import are also upregulated to ensure proper import of essential protein homeostatic machinery (Xin et al., 2020). UPR^MT^ targets are not only limited to protein homeostasis, as genes involved in immunity (Pellegrino et al., 2014), autophagy (Haeussler et al., 2020), and xenobiotic stress (Nargund et al., 2012; Nargund et al., 2015) are also induced. Moreover, direct applications of stress to different mitochondrial processes with drugs results in vast differences in transcriptional response (Quirós et al., 2017), thus highlighting the complexities of mitochondrial stress signaling and regulation (refer to (Bar-Ziv et al., 2020a) for a more thorough review). Here, we focus primarily on the impact of UPR^MT^ on aging in the model organism, *C. elegans*.

### UPR^MT^ and Aging: Mitochondrial Hormesis Through Inhibition of the Electron Transport Chain

The UPR^MT^ was first used to describe a transcriptional response whereby loss of the mitochondrial genome led to increased expression of mitochondrial chaperones. This process was originally identified in mammalian cells and was named after its presumed effects on mitochondrial protein folding (Martinus et al., 1996). Further studies identified additional targets for UPR^MT^ activation, which include ROS detoxification, mitochondrial import, and glycolysis (Zhao et al., 2002). Since then, it has been heavily studied in various model systems, most notably in *C. elegans*, as a beneficial response to stress. In this model coined “mitohormesis”, exposure to low grade stress to the mitochondria results in the induction of an adaptive program that can benefit both lifespan and healthspan (Ristow and Zarse 2010). In *C. elegans*, the phenomenon of mitohormesis was first identified in a large-scale RNAi screen of genes found on chromosome I. In this study, RNAi knockdown of three components of the mitochondrial ETC, mitochondrial ATP synthase (*atp-1*), NADH/ubiquinone oxidoreductase (*nuo-2*), and cytochrome c reductase (*cco-1/cox-5b*) all resulted in reduced body size and extended lifespan (Dillin et al., 2002). A similar RNAi screen of nearly all the genes on chromosomes I and II consistently found that knockdown of a large number of mitochondrial genes extended lifespan, including cytochrome C oxidase VIIc (*cox-7c*), mitochondrial ribosomal protein L47 (*mrpl-47*), and the mitochondrial solute carrier family 25 member 32 (*slc-25A32*) (Lee et al., 2003). This study also performed a classical forward genetic screen and identified a mitochondrial leucyl-tRNA synthetase (*lrs-2*), which similarly extends lifespan when disrupted. Since then, multiple perturbations to mitochondrial function have demonstrated a positive impact on lifespan: mitochondrial ribosomal protein knockdown (Houtkooper et al., 2013), perturbing mitochondrial dynamics in favor of mitochondrial fusion (Chaudhari and Kipreos 2017), mitochondrial genome impairment (Tsang and Lemire 2002), and many others reviewed in (Munkácsy and Rea 2014).

An intriguing phenomenon in *C. elegans* is that there is a specific temporal requirement during development to enact the positive benefits of mitohormesis. That is, animals exposed to RNAi knockdown of ETC components during development exhibited lifespan extension, even if normal levels of ETC component expression were restored at adulthood (Li et al., 2021). Conversely, reducing levels of ETC components in adulthood had no impact on lifespan. These data suggested that a long-lasting signature exists to elicit lifespan extension following mitochondrial dysfunction. This specific signal is initiated during the L3/L4 larval stage of development (Dillin et al., 2002; Rea et al., 2007). Interestingly, this phenomenon also had a dose-dependent effect. Titrating the RNAi of five genes encoding mitochondrial proteins *atp-3*, *nuo-2*, *isp-1*, *cco-1*, and *frh-1* showed that there was a consistent three-phase lifespan response: low levels of knockdown had no effect, but as expression reduced, lifespan extension lengthened until at the highest level of knockdown, lifespan actually shortened for some conditions (Rea et al., 2007).

### UPR^MT^ and Aging: Chromatin Changes Result in Long-Term Effects

The most obvious response to mitochondrial stress is the massive and persistent restructuring of gene expression, which is a hallmark of UPR^MT^ activation. In *C. elegans*, this process is mediated by the converted effort of the transcriptional regulators ATFS-1, DVE-1, and UBL-5 (Benedetti et al., 2006; Haynes et al., 2007; Nargund et al., 2012). However, the existence of a long-term hormesis suggested that a more permanent signature must exist upon exposure to mitochondrial stress. One study identified the methyltransferase MET-2 and a nuclear cofactor LIN-65 are required for UPR^MT^ activation and lifespan extension found in animals with exposure to mitochondrial stress (*cco-1* knockdown or exposure to aggregation prone polyQ described further below) (Tian et al., 2016). Specifically, 1,264 of the 1,312 genes differentially expressed under *cco-1* knockdown were dependent on the functional activity of either MET-2 or LIN-65. During mitochondrial stress, MET-2 produces H3K9me1/2. Nuclear localization of LIN-65 then deposits H3K9me2 subunits onto the chromatin, titrating DVE-1 to loose regions of the chromatin and allowing for sustained ATFS-1 localization at these loci. Thus, loss of any of these factors under stress could result in decreased UPR^MT^ activity and loss of lifespan extension.

A complementary study identified another chromatin modifier as a critical regulator of UPR^MT^-induced longevity (Merkwirth et al., 2016). This study performed a screen to identify genes required for the lifespan extension found in *cco-1* knockdown animals and identified the gene encoding the histone demethylase, *jmjd-1.2*. Knockdown of *jmjd-1.2* decreased lifespan and suppressed cco-1 induced lifespan extension, similar to *met-2* and *lin-65*. Importantly, overexpression of *jmjd-1.2* was sufficient to induce UPR^MT^ and promote longevity. This was the first study that showed that hyperactivation of UPR^MT^ in the absence of stress was sufficient to extend lifespan. Because JMJD-1.2 functions downstream of mitochondrial stress, its overexpression bypasses the need to cause damage to the mitochondria to induce UPR^MT^.

### UPR^MT^ and Aging: A Conundrum and Paradox

An unaddressed conundrum is that the targets of UPR^MT^ include a large number of chaperones and other protein processing components that must be imported into the mitochondria to serve their function to restore organellar homeostasis. However, the dogma for UPR^MT^ activation is that decreased mitochondrial membrane potential drives the mitochondria-to-nuclear signaling by preferential localization of ATFS-1 to the nucleus due to failed entry in the mitochondria. How then do proteins get imported into the mitochondria when membrane potential is compromised? In an exciting piece of work, this paradox was addressed by finding that the induction of UPR^MT^ can actually increase mitochondrial import, despite a loss of membrane potential (Xin et al., 2020). The efficiency of import is increased by upregulating components of the mitochondrial import machinery, including *timm-17*, *timm-23*, *tomm-20*, *tomm-22*, and *tomm-40*. In addition, because ATFS-1 has a weak MTS, it continues to fail to enter the mitochondria and can continue to drive UPR^MT^ induction until mitochondrial function is fully restored.

Interestingly, lifespan was never reportedly increased in response to knockdown of complex II subunits (Ichimiya et al., 2002; Rea et al., 2007; Kuang and Ebert 2012). In addition, a screen for novel regulators of UPR^MT^ identified 19 genes that when knocked down induce UPR^MT^ but showed no correlation for lifespan extension. In fact, 6 out of the 19 RNAi conditions actually decreased lifespan, while 3 showed no significant effect (Bennett et al., 2014). Notably, all the genes that decreased lifespan were important for mitochondrial import, which a previous study has implicated as necessary for any beneficial effects of UPR^MT^ (Xin et al., 2020). Thus, it is entirely possible that when mitochondrial import is reduced, there is robust activation of UPR^MT^ without a beneficial impact on organismal health, which would likely require import of newly synthesized mitochondrial protein homeostasis machinery. However, a striking finding in the study was that the lifespan extension found in several conditions of mitochondrial stress induction (RNAi knockdown of transaldolase *tald-1*, mitochondrial transmembrane protein *letm-1*, *cco-1*, and *isp-1*) were all independent of *atfs-1*. Moreover, constitutive activation of *atfs-1* did not improve lifespan, but instead shortened it, despite measurable activation of UPR^MT^ (Bennett et al., 2014), although it is unclear whether atfs-1 activation may cause unexpected and detrimental consequences to mitochondrial quality (Shpilka et al., 2021). In another study, loss of *atfs-1* during adulthood did not affect lifespan extension of *clk-1*, *isp-1*, or *nuo-6* loss of function animals (Wu et al., 2018), consistent with previous findings that the beneficial impact of mitochondrial stress may be important only during development (Durieux et al., 2011). However, deletion of *atfs-1* during development resulted in defects in growth in *clk-1* and *isp-1* loss of function animals, although it did suppress lifespan of *nuo-6* mutants. Overall, these conflicting data argued against the longstanding model that activation of UPR^MT^ improves longevity.

Taken together, it is clear and consistent across numerous studies that mitohormesis is evident: exposure to mitochondrial dysfunction can indeed activate a protective pathway that can promote lifespan extension. However, whether this protective pathway can be fully ascribed to the currently defined model of UPR^MT^ is still up for debate. The response to mitochondrial stress in human cells is complex and context-dependent, as diverse drugs that target different components of the mitochondria show little overlap in transcriptional response (Quirós et al., 2017). Similarly, it is likely that activation of UPR^MT^ in *C. elegans* is equally context dependent, and a broader definition of UPR^MT^ beyond induction of a small subset of chaperones (e.g., *hsp-6* and *hsp-60*) is necessary. Perhaps a more thorough investigation of specific UPR^MT^ targets downstream of multiple transcriptional regulators–and not just ATFS-1–is essential to understand the nuances between seemingly “UPR^MT^” dependent and independent mechanisms of lifespan extension downstream of mitochondrial dysfunction. For example, many other pathways including activation of hypoxia inducible factor HIF-1 (Kourtis et al., 2012), homeobox protein CEH-24 (Walter et al., 2011), AMP-activated protein kinase AAK-2 (Curtis et al., 2006), and the p53 homolog CEP-1 (Ventura et al., 2009); and the integrated stress response (ISR) critical for responding to mitochondrial stress in higher mammals (reviewed in (Anderson and Haynes 2020)) have all been implicated in longevity in response to mitochondrial stress in *C. elegans*. In addition, there are also gene-diet interactions that alter the impact of UPR^MT^ on longevity (Amin et al., 2020). Thus, in its current state, it is clear that a single mechanism cannot describe all the mitochondrial longevity paradigms and more expansive studies are required to better understand this complex phenomenon.

### Neuronal Transmission of UPR^MT^

Under conditions of stress, multicellular organisms must coordinate a systemic response across diverse cell types, all with their own unique energetic and metabolic demands. Mitochondrial number, activity, protein composition, morphology, and mtDNA contents can all vary across different tissues (Leary et al., 1998), and thus responses to mitochondrial stress are unique in each cell type. In *C. elegans*, exposure to mitochondrial stress specifically in the intestine or neurons results in a significant increase in lifespan, whereas exposure of muscle cells to stress had the opposite effect and shortened lifespan (Durieux et al., 2011), highlighting the significance of context-specific benefits of UPR^MT^ activation. Importantly, applying mitochondrial stress to neurons was sufficient to induce systemic activation of UPR^MT^ due to a neuron-to-periphery cell non-autonomous response, which was critical for lifespan extension (Figure 2B) (Leary et al., 1998). This early paradigm of non-autonomous UPR^MT^ involved a “Mjolnor hammer” approach where perturbation of ETC function via *cco-1* knockdown caused severe mitochondrial stress in neurons. However, since then, more physiologically relevant stressors confirmed these findings where overexpression of polyQ40 (and larger polyQ repeats) (Berendzen et al., 2016) or expression of ROS-producing KillerRed fluorescent protein (Shao et al., 2016) specifically in neurons robustly induced UPR^MT^ in peripheral cells. Interestingly, overexpression of amyloid *ß* associated with Alzheimer’s disease or mutant TDP-43 associated with amyotrophic lateral sclerosis in neurons failed to induce peripheral UPR^MT^ activation, suggesting that this neuronal stress signaling paradigm was also context specific (Shao et al., 2016). Importantly, neurons can transmit UPR^MT^ signals to the periphery in the absence of stress, as overexpression of *jmjd-1.2* solely in neurons was also able to induce this non-autonomous UPR^MT^ signal and was sufficient to promote longevity (Merkwirth et al., 2016).

The phenomenon whereby neurons can transmit stress signals to the rest of the body is not unique to UPR^MT^, and has also been described for UPR^ER^ (Taylor and Dillin 2013) and the HSR (Prahlad et al., 2008b; Higuchi-Sanabria et al., 2018b) (see individual sections), although the mechanisms driving these responses seem to be distinct. While UPR^MT^ (Berendzen et al., 2016), UPR^ER^ (Higuchi-Sanabria et al., 2020), and HSR (Tatum et al., 2015) all seem to utilize serotonergic circuits, Wnt signaling was also identified to be critical for transmitting the UPR^MT^ signal (Zhang et al., 2018). Specifically, knockdown of *mig-14*, *dpy-23*, and *mig-1*, critical components in receiving and internalizing Wnt signals completely abolished UPR^MT^ activation downstream of neuronal polyQ40 expression. Most importantly, overexpression of the gene encoding the secreted Wnt ligand *egl-20* specifically in neurons was sufficient to drive systemic UPR^MT^ activation and extend lifespan. Collectively, these findings have led to the conclusion that there exist not one, but two secreted mitokines from neurons that drive mitochondrial stress signaling, serotonin and Wnt. Perhaps most intriguing was the finding that this induced systemic UPR^MT^ downstream of neuronal mitochondrial perturbations were transmitted to offspring over multiple generations in *C. elegans*. Specifically, neuronal expression of polyQ40 resulted in a transgenerational induction of UPR^MT^ that was observed more than 50 generations out upon loss of the neuronal polyQ40 transgene. This transmission of UPR^MT^ to offspring was through a Wnt-dependent elevation of mtDNA levels across generations (Zhang et al., 2021).

The capacity of cells to transmit mitochondrial stress signals to other cells is not a unique feature of neurons. In fact, germline-specific loss of the cytochrome c ortholog, *cyc-2.1* initiates a non-autonomous response that activates UPR^MT^ and AMPK in the intestine, which results in a robust lifespan extension. Importantly, this lifespan extension was dependent on ATFS-1, which drives DRP-1-mediated mitochondrial fragmentation (Lan et al., 2019). Decreased protein homeostasis in the germline–defined in this context as increased aggregation of PGL-1, an RNA-binding protein involved in P granule formation in the germline–also resulted in a non-autonomous activation of UPR^MT^ in the soma, providing evidence that this germline-to-soma transmission of UPR^MT^ could be a general response to germline stress. Increased PGL-1 aggregation resulted in a significant decrease in mitochondrial protein levels in the germline, which resulted in a Wnt-dependent transmission of mitochondrial stress signals to the soma. Specifically, PGL-1 aggregation in the germline resulted in an EGL-20 (Wnt ligand) and MIG-1 (Wnt receptor)-dependent mitochondrial fragmentation and UPR^MT^ induction in the soma (Calculli et al., 2021). However, it is still unclear whether this somatic UPR^MT^ induction is beneficial to organismal health and lifespan.

### Perspectives and Concluding Remarks

Perhaps the most controversial point of UPR^MT^ on aging is whether activation of UPR^MT^ directly correlates with aging. As described above, knockdown of complex II genes never exhibited lifespan extension, while other methods to induce UPR^MT^ have inconsistent impacts on lifespan (Bennett et al., 2014). The most obvious hypothesis is that there are varying degrees of mitochondrial stress, whereby only those conditions that induce sufficient mitochondrial stress to activate UPR^MT^ without causing irreversible damage can promote hormesis and extend lifespan. In contrast, causing too much damage would be detrimental, regardless of whether UPR^MT^ is activated or not. Another plausible explanation is that the methods to measure UPR^MT^ activation are not sufficient: most studies rely on artificial transcriptional reporters, such as the overexpression of the *hsp-6p::GFP* reporter. As an overexpressed system, it is possible that *hsp-6p::GFP* exaggerates the intensity of UPR^MT^ activation, which is clear in some reports where western blots and qPCR show markedly lower *hsp-6* induction than the reporter (Berendzen et al., 2016). Thus, it is possible that a more thorough investigation of UPR^MT^ activation (e.g., survey of more gene targets, measurements of nuclear localization of UPR^MT^ regulators like DVE-1::GFP, measurements of chromatin compaction, etc.) is essential for understanding the true impact of UPR^MT^ on longevity.

Additionally, it would be of great interest to determine whether other cell types can initiate non-autonomous mitochondrial stress signatures. In flies, mitochondrial perturbations through dysfunction of complex I specifically in muscle results also results in impairment of mitochondrial function in the body fat (Song et al., 2017). Moreover, in mice, muscle-specific deletion of a critical autophagy gene Atf7 results in increased secretion of Fgf21, which increased resistance of these animals to diet-induced obesity (Kim et al., 2013). Importantly, Fgf21 serves as an endocrine signal to elicit ATF3-, ATF-4, and ATF-5 dependent ISR and UPR^MT^ (Forsström et al., 2019). Thus, it is entirely possible that other cell types in *C. elegans* also have the capacity to promote systemic UPR^MT^ through non-autonomous signaling.

Finally, while we survey the impact of UPR^MT^ on longevity in *C. elegans*, it is of critical importance to put into perspective the translatability of these findings to mammalian systems. One report argued that ATF5, the predicted mammalian homologue of ATFS-1, is critical for mitochondrial quality control (Fiorese et al., 2016). However, large-scale -omics-based approaches showed that the major responses to various drugs that target mitochondrial processes involved ATF4, which activates the ISR (Quirós et al., 2017). Furthermore, studies of human cells in more physiologically similar matrices (softer, 400 Pa hydrogels compared to the ∼3 GPa of polystyrene) highlighted the major involvement of HSF1 and NRF2 in mitochondrial homeostasis in cancer cells (Tharp et al., 2021). Thus, it is still unclear how UPR^MT^ is regulated in mammalian systems, how ISR, UPR^MT^, HSF1, and NRF2 either simultaneously or independently coordinate aspects of mitochondrial homeostasis, and ultimately, what impact–if any–these mechanisms have on mammalian longevity.

### The Endoplasmic Reticulum Unfolded Protein Response

The endoplasmic reticulum (ER) is a complex multi-faceted organelle. While maintaining a contiguous membrane with the nucleus, the ER functions in protein folding, lipid synthesis, lipid droplet formation, and calcium storage (Fagone and Jackowski 2009; Benham 2012; Pol et al., 2014; Raffaello et al., 2016). The ER must also coordinate these responsibilities with other cellular organelles to traffic transmembrane proteins, lipids, and calcium. Therefore, a functional ER is essential to maintaining a healthy cellular status. Perturbations in ER protein quality control have been implicated in age-related diseases like Alzheimer’s disease and Parkinson’s disease (Hartl 2017), while dysregulation of lipid synthesis has been associated with diabetes and cardiovascular disease (Chaurasia and Summers 2015; Sletten et al., 2018). Activation of the unfolded protein response of the ER (UPR^ER^), a transcriptional program activated upon exposure to ER stress, is often correlated with the development of these diseases as well as cancer (Clarke et al., 2014; Zhang et al., 2017). Interestingly, studies have also shown that genetic activation of the most conserved branch of the UPR^ER^ can instead result in health benefits and an increased lifespan (Taylor and Dillin 2013; Frakes et al., 2020). These opposing phenotypes highlight the complexity and importance of understanding the role that the UPR^ER^ plays in disease and aging.

**FIGURE 3:**
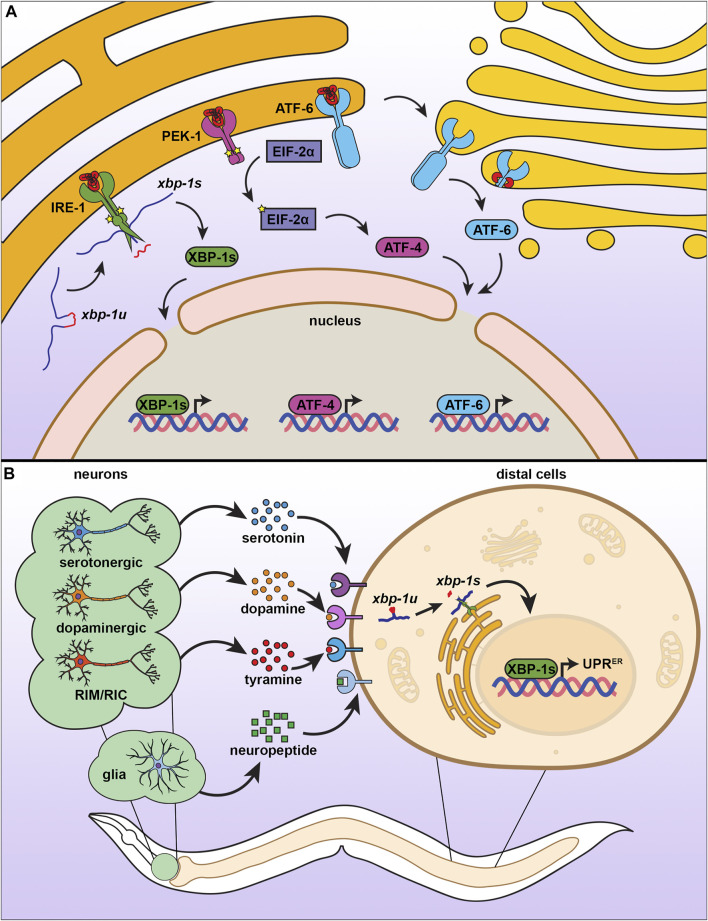
The endoplasmic reticulum unfolded protein response. **(A)** There are three branches of UPR^ER^, each consisting of an ER transmembrane protein with different mechanisms of action. When IRE-1 senses misfolded proteins, it homodimerizes, autophosphorylates, and promotes splicing of *xbp-1u* to *xbp-1s*, which can then be translated into the transcription factor XBP-1s, which activates UPR^ER^. Similar to IRE-1, PEK-1 undergoes oligomerization, which induces eIF2α phosphorylation and activation of ATF4 to inhibit global translation. ATF6 is activated by proteolytic cleavage under ER stress, which causes translocation to the Golgi for further processing, resulting in a transcriptionally active ATF6 that promotes UPR^ER^. **(B)** The UPR^ER^ is communicated in a nonautonomous manner through multiple neuronal subtypes and through glial cells. Serotonergic, dopaminergic, and RIM/RIC neurons signal to peripheral cells using specific neurotransmitters, whereas glial cells communicate through neuropeptide signaling. While the exact identify of receptors involved have not yet been fully characterized, distal cells respond by promoting IRE-1-dependent splicing of xbp-1s to induce the UPR^ER^.

Similar to the mammalian ER, the *C. elegans* UPR^ER^ is composed of three signaling branches, each branch signaling from a unique transmembrane sensor (Figure 3A) (Shen et al., 2001). Initially discovered through a genetic screen for mutants that failed to induce an Unfolded Protein Response Element (UPRE) reporter in yeast, the most conserved of the these UPR^ER^ sensors is inositol-requiring enzyme 1 (*ire-1*) (Cox et al., 1993; Aragón et al., 2009). The IRE-1 protein is composed of an N-terminal ER luminal domain that is linked by a single-pass transmembrane domain to its cytosolic portion. The cytosolic side of IRE-1 contains both a kinase domain and an RNAse domain (Adams et al., 2019). In unstressed conditions, IRE-1 is bound to the chaperone HSP-4 (HSP70/BiP) at the luminal domain, which maintains IRE-1 as a monomer (Amin-Wetzel et al., 2017). Upon unfolded protein stress, HSP-4 is titrated away from IRE-1, freeing the luminal domain to directly bind unfolded proteins and dimerize (Zhou et al., 2006). Dimerized IRE-1 undergoes autophosphorylation, which activates its RNAse domain (Prischi et al., 2014), resulting in non-canonical splicing of the *xbp-1* mRNA to allow synthesis of the active transcription factor, XBP-1s (Calfon et al., 2002). XBP-1s can then enter the nucleus and induce expression of genes aimed at mitigating the stress on the ER, including those involved in protein quality control, secretion, and lipid metabolism (Shen et al., 2005).

In addition to the induction of ER homeostasis genes through activating *xbp-1s*, IRE-1 also plays a functional role in the degradation of mRNAs in a process known as Regulated Ire1 Dependent Decay (RIDD) (Tam et al., 2014). Targets of RIDD generally include proteins with signal peptides and transmembrane domains, or secretory proteins whose decreased translation is expected to reduce the protein folding burden of the ER (Hollien and Weissman 2006; Lee et al., 2011; Tsuru et al., 2013). Mammalian IRE-1 can also interact with TNF Receptor Associated Factor 2 (TRAF2) and activate the c-Jun N-terminal kinase (JNK) pathway (Urano et al., 2000). Activation of JNK signaling can promote cell death, while inhibiting downstream activation of JNK can promote cell survival under ER stress (Nishitoh et al., 2002; Verma and Datta 2010).

The UPR^ER^ sensors of other two branches of the UPR^ER^ are the protein kinase R (PKR)-like endoplasmic reticulum kinase (*pek-1*) and the activating transcription factor 6 (*atf-6*). Similar to IRE-1, both PEK-1 and ATF-6 contain a luminal domain that binds unfolded proteins, a transmembrane domain, and a cytosolic domain. However, they differ in the functions of the cytosolic domain (Schröder and Kaufman 2005). While PEK-1 also contains a kinase domain on its cytosolic side, it does not possess RNAse function (Cui et al., 2011). Instead, activation of PEK-1 results in the phosphorylation of the alpha subunit of eukaryotic initiation factor (*eif-2A*) to reduce global translation, but favors translation of the transcription factor ATF-4 to induce expression of genes to aid in mitigation of the ER stress (Han et al., 2013; Ma and Hendershot 2003; B’chir et al., 2013). The final branch of the UPR^ER^ has an entirely different mechanism of action and involves the cytosolic domain of the ATF-6 protein, which contains a leucine zipper family transcription factor (Schröder and Kaufman 2005). Upon the accumulation of unfolded proteins, the luminal domain of ATF-6 loses its association with ER-resident chaperones, which results in its translocation to the Golgi (Stauffer et al., 2020) where it is further processed (Haze et al., 1999; Ye et al., 2000). The ATF-6 transcription factor then enters the nucleus to promote expression of UPR^ER^ target genes (Shoulders et al., 2013; Stauffer et al., 2020).

### UPR^ER^ During Stress

Like in most organisms, the IRE-1 branch of the UPR^ER^ is the most heavily studied branch in *C. elegans*. The IRE-1 branch is primarily responsible for the induction of genes encoding ER resident chaperones, including *hsp-4* and *hsp-3* (Shen et al., 2001). As such, the transcriptional reporter for *hsp-4* has become one of the most common and robust methods of monitoring UPR^ER^ activation within the nematode (Calfon et al., 2002; Bar-Ziv et al., 2020b). However, it is becoming increasingly clear that chaperone induction and protein homeostasis are not the only targets of UPR^ER^, as other critical processes are also induced including immune response, lysosomal function and autophagy, and lipid homeostasis. The UPR^ER^ also plays an important role in development in *C. elegans* and the simultaneous loss of multiple branches results in developmental arrest of animals (Shen et al., 2005). Importantly, the IRE-1 branch of UPR^ER^ is critical for the exit from cellular quiescence (Roux et al., 2016). Newly hatched nematodes that enter L1 arrest due to the absence of food can exit this quiescent state to resume normal development and a normal lifespan upon the introduction of food. Larvae that maintain the L1 arrest for extended periods require ire-1 to recover from this state and resume development. The mRNA degradation function of IRE-1 has also been shown to regulate cellular fate decisions in *C. elegans*. Activation of IRE-1 within tumorous germline cells stimulates differentiation that halts progression of the tumor growth (Levi-Ferber et al., 2015; Levi-Ferber et al., 2021). This function is also conserved in mammals, *drosophila*, and yeast, where the UPR^ER^ has also been found to be required for normal development of various cell types (Reimold et al., 2001; Jung et al., 2016; Xu et al., 2016; Hillary and FitzGerald 2018; Kroeger et al., 2018).

Dysregulation of the UPR^ER^ is a common feature in many diseases, including neurodegeneration, metabolic disease, and cancer, and has been shown to decline in function during aging. In *C. elegans*, the capacity to induce IRE-1 and XBP-1 mediated UPR^ER^ fundamentally declines during aging, which results in increased sensitivity to ER stress (Taylor and Dillin 2013). A similar phenomenon is seen in aged mice where expression of ER quality control genes show marked decline in the brain (Naidoo et al., 2008; Nuss et al., 2008). The loss of UPR^ER^ function during aging can lead to the accumulation of damaged and aggregated proteins, which results in the physiological consequences of aging including increased proteotoxicity and cell death (Estébanez et al., 2018).

Although the UPR^ER^ is an adaptive and beneficial mechanism involved in clearing and mitigating damage under conditions of stress, sustained and unresolved UPR^ER^ can result in the activation of apoptosis. As such, chronic and irreversible UPR^ER^ is actually a hallmark for several disease, including neurodegeneration. Unresolved ER stress can activate pro-apoptotic machinery, including the C/EBP homologous protein CHOP, which downregulates anti-apoptotic factors, like B-cell lymphoma 2 (BCL2) (Chauve et al., 2021). Under certain conditions, ER stress can also promote MAPK signaling, including ERK1/2, which can be a pro-cancer signal, which makes inhibiting UPR^ER^ a potential therapeutic intervention for these cancers (Jiang et al., 2007; Beck et al., 2013). In addition, activation of the UPR^ER^ can also promote an inflammatory signaling cascade, including cytokine release, which can be correlated with common inflammatory disease including diabetes, atherosclerosis, and inflammatory bowel disease (Zhang and Zhang 2012; Coleman and Haller 2019). These studies suggest a potentially negative role for UPR^ER^ in organismal health and disease pathology. Indeed, studies in *C. elegans* have also revealed a potentially negative role for the UPR^ER^, whereby whole-organism overexpression of xbp-1s is not beneficial to longevity and muscle-specific overexpression actually reduces lifespan (Taylor and Dillin 2013). Thus, while it is clear that the connection between UPR^ER^ and longevity are complex, myriad studies in *C. elegans* are in agreement that UPR^ER^ in the nervous system can be highly beneficial to organismal health. Thus, we focus on how UPR^ER^ function can impact the aging process in *C. elegans*, which primarily involves non-autonomous communication of ER stress signals through neural cells (Figure 3B).

### UPR^ER^ and Aging: Neuronal Transmission of UPR^ER^

Multicellular organisms coordinate systemic changes for development and metabolism through secretion of factors from the nervous system (e.g., neurotransmitter, hormones). Indeed, perceived ER stress in neural cells results in secretion of signals to orchestrate an organismal response improve ER homeostasis, ER stress resilience, and longevity. Specifically, overexpression of the spliced *xbp-1s* transcription factor within neurons or amphid sheath glia of *C. elegans* results in activation of the UPR^ER^ not only within the neurons and glia themselves, but also within distal intestinal cells (Taylor and Dillin 2013; Frakes et al., 2020). This non-autonomous induction of the UPR^ER^ results in increased stress resilience and longevity. Interestingly, the mechanisms whereby neurons and glia coordinate this organism-wide response differ: non-autonomous signaling through neurons is dependent on the release of small clear vesicles (SCVs) through UNC-13, such that loss of *unc-13* results in elimination of the beneficial effects of neuronal *xbp-1s* overexpression (Taylor and Dillin 2013); in contrast, non-autonomous signaling through glia are dependent on release of neuropeptides through the release of dense core vesicles mediated by UNC-31 and neuropeptide processing by EGL-3 (Frakes et al., 2020). The distinction between the two origins of the UPR^ER^ signal is further highlighted by the partially additive increase in lifespan that is observed when *xbp-1s* is overexpressed in both types of neural cells. As neurons and glia have a well-established relationship, it would be likely that both paradigms share some form of communication or mechanism to extend lifespan, though current research has not yet clarified whether direct communication between neurons and glia exist in this paradigm.

In its original study, the beneficial effects of neuronal UPR^ER^ were ascribed to the upregulation of chaperones, which resulted in increased protein homeostasis and ER stress resilience. Since then, further studies found that neuronal UPR^ER^ also improved lipid metabolism. Lipid staining and lipidomic analysis revealed a decrease in neutral lipid stores, while concurrently showing an increase in monounsaturated fatty acids, including oleic acid (Imanikia et al., 2019a). In fact, supplementation of oleic acid was sufficient to increase the lifespan of wild-type and *xbp-1* deficient animals but did not further extend the lifespan of xbp-1s overexpressing animals, providing further evidence that the beneficial effects of neuronal *xbp-1s* could be at least partially ascribed to changes in lipid profiles. Interestingly, oleic acid supplementation also provided protection against the effects of proteotoxic polyQ40 and α-Aß proteins, suggesting that improving lipid metabolism in the ER could also impact protein homeostasis, though the mechanism whereby this happens is still unexplored (Imanikia et al., 2019a). Importantly, this study ascribed the primary mechanism of lipid remodeling to the desaturases FAT-6 and FAT-7, which metabolized neutral lipid stores into oleic acid to promote organismal health.

The contribution of lipid metabolism to the beneficial effects of non-autonomous UPR^ER^ phenotypes was further indicated by the expansion of lysosomes and increased lipophagy found in these animals (Imanikia et al., 2019b; Daniele et al., 2020a). Neuronal *xbp-1s* overexpression resulted in a significant expansion of lysosomes and increased expression of lysosomal lipases. Changes to lysosomal activity were essential for the beneficial effects of neuronal *xbp-1s* as knockdown of the *C. elegans* homolog to mammalian TFEB, *hlh-30*, was sufficient to suppress the lifespan extension of these animals (Imanikia et al., 2019b). This is in direct agreement with another study that found that HPL-2, a chromatin modifying protein, promotes autophagy to increase ER stress resilience (Kozlowski et al., 2014). Further, transcriptional profiling of worms deficient in phosphatidylcholine (PC) synthesis–which causes ER stress through lipid dysregulation–also induced autophagy in an IRE-1/XB-1-dependent manner (Koh et al., 2018). This is highly similar to a process previously described in yeast, where inhibition of PC biosynthesis activates microlipophagy downstream of UPR^ER^ (Vevea et al., 2015). Indeed, in *C. elegans*, promoting lipophagy by overexpression of *ehbp-1*, a core component of the conserved RME-1/RAB-10/EHBP-1 lipophagy complex, was sufficient to drive lipid remodeling and lifespan extension (Daniele et al., 2020a). Importantly, this beneficial effect of lipophagy downstream of neuronal UPR^ER^ was independent from its canonical role in protein homeostasis through chaperones.

A subsequent study found that the chaperone induction and altered lipid metabolism downstream of non-autonomous UPR^ER^ signaling was in part due to signaling from distinct subsets of neurons (Higuchi-Sanabria et al., 2020). Overexpression of xbp-1s within dopaminergic neurons was sufficient to drive EHBP-1 regulated lipid metabolism, while overexpression in serotonergic neurons induced expression of ER chaperones to promote protein homeostasis. Both sources of non-autonomous UPR^ER^ signals independently promoted organismal health and lifespan, and their effects were observed to be additive, suggesting an intricate model whereby neurons can differ in their capacity to elicit a peripheral response through unique stress signals. Indeed, a separate genetic screen to identify neurotransmitters involved in neuronal UPR^ER^ signals failed to identify dopamine or serotonin signaling, and instead found that tyramine synthesis was essential for transmitting UPR^ER^ from neurons to the intestine (Özbey et al., 2020). Overexpression of *xbp-1s* within the tyraminergic neurons was sufficient to induce the UPR^ER^ within the intestine and stimulate changes to the animal’s feeding behavior and brood size. While dopaminergic, serotonergic, and tyraminergic neurons have now been shown to contribute to the non-autonomous UPR^ER^, whether these neurons directly communicate with each other or whether other subtypes or specific neurons may also contribute to–or potentially hinder–the longevity phenotypes of the non-autonomous UPR^ER^ remains to be known.

Neuronal transmission of an ER stress response is not limited to the genetic models observed in *C. elegans*. When Xbp1s is overexpressed in POMC neurons of mice, a similar non-autonomous activation of UPR^ER^ exists in peripheral cells and beneficially impacts metabolic physiology (e.g., improved glucose homeostasis, increased insulin sensitivity, and protection against high-fat diet induced obesity) (Williams et al., 2014). Importantly, a similar process can occur with naturally occurring stimuli whereby sensory perception of food can result in activation of POMC neurons to activate UPR^ER^ within the liver to prepare for incoming nourishment (Brandt et al., 2018). This induction resulted in increased lipid synthesis and remodeling of the ER, likely to prime the liver for its roles in processing the nutrients. However, how this nonautonomous signaling impacts aging or longevity has not been confirmed, still making a conclusive correlation between UPR^ER^ activity and lifespan difficult.

### Perspectives and Concluding Remarks

As aptly named, the historical function ascribed to the UPR^ER^ is to promote protein homeostasis, though it has become increasingly clear that the UPR^ER^ regulates many critical functions outside of protein quality control, including autophagy and lipid homeostasis as described briefly above. One important question is how these functional roles overlap. For example, it isn’t difficult to ascertain how increased lysosomal function can promote autophagy to clear damaged proteins, ultimately resulting in increased protein homeostasis. However, increased lipid metabolism downstream of nonautonomous UPR^ER^ signals also resulted in increased clearance of protein aggregates (Imanikia et al., 2019a) and increased resistance to protein misfolding stress (Higuchi-Sanabria et al., 2020). It is entirely possible that improved lipid metabolism simply increases general organismal health, making animals more resilient to all sources of stress or increases the resources available for mitigating damage. Alternatively, it is possible that improved lipid homeostasis can actually have direct impacts on protein homeostasis. For example, increased lipid metabolism can increase secretory capacity of the ER (Daniele et al., 2020b), and it is possible that damaged proteins can be secreted to external environments, such as the pseudocoelom in C.*elegans*, where it will cause less damage.

Beyond the beneficial roles of the UPR^ER^, unresolved UPR^ER^ signaling can be detrimental. For example, we briefly discussed how chronic UPR^ER^ can cause apoptosis in mammalian cells, and overexpression of HAC1s, the *S. cerevisiae* homolog of *xbp-1*, can perturb cell cycle progression (Sopko et al., 2006). However, promoting UPR^ER^ in *C. elegans* seems to promote organismal health and extend lifespan, which can be due to the post-mitotic nature of the adult worm, which is not deterred by cell cycle or apoptosis machinery. Indeed, ectopic activation of UPR^ER^ has negative consequences in the germline, the few actively dividing cells of the adult worm, whereby neuronal UPR^ER^ causes decreased fecundity and brood size (Özbey et al., 2020). However, this differentiation between actively dividing versus post-mitotic cells still does not explain the negative impact of *xbp-1s* overexpression in muscle cells of *C. elegans* (Taylor and Dillin 2013), as these cells are also post-mitotic. One plausible explanation is that increased UPR^ER^ causes lipid depletion, which may be detrimental to the highly energy-demanding muscle cells.

A final thought that we find important to mention is to understand the multi-faceted response of UPR^ER^. With the increasing number of mechanistic pathways being ascribed to UPR^ER^ activation, how then does a cell coordinate these downstream pathways? For example, serotonergic and dopaminergic signals elicit two different responses in peripheral cells: induction of chaperones and lipid regulatory enzymes, respectively. How does one transcription factor, XBP-1s, coordinate two distinct responses? It is possible that other cofactors titrate XBP-1s to its appropriate targets; for example, TFEB/HLH-30 is required for the changes to lysosomal genes downstream of XBP-1s activation, suggesting that interaction of TFEB/XBP-1s is important for lysosomal genes. Still to be identified are what other XBP-1s interactors exist to titrate XBP-1s specifically to other target pathways, including lipid regulation, ERAD, or chaperones.

### Impact of Cellular Stress Responses on Other Compartments

Perhaps one historically overlooked issue in the field of stress biology is in its often single focus: generally, stress responses are studied with a focus on the single organelle that it impacts. However, it is becoming increasingly clear that activation or perturbation of stress responses elicit pleiotropic changes that alter multiple systems.

### Mitochondria-ER Interactions During Stress

The functional state of the mitochondria directly impacts the state of other organelles, and thus it is unsurprising that UPR^MT^ function has ramifications on other cellular processes. For example, ER-mitochondria contact sites (ERMCS) allow for the exchange of proteins, metabolites, ions, and lipids between the organelles and are critical in maintaining cellular homeostasis (Xu et al., 2020). In HeLa cells, mitochondrial stress induced by doxycycline exposure can increase ERMCS (Lopez-Crisosto et al., 2021), potentially through UPR^MT^ activation. Increased ERMCS can also result in creased lifespan in *drosophila* (Garrido-Maraver et al., 2020), opening up the possibility that UPR^MT^ activation may potentially increase lifespan through effects on ERMCS. One important function of ERMCS is CA^2+^ homeostasis regulated by ER-to-mitochondria CA^2+^ transfer. In *C. elegans*, the UPR^ER^ regulator, ATF-6 regulates lifespan through modulation of ER-mitochondria calcium transport. As *C. elegans* age, ATF-6 activity increases, resulting in activation of UPR^ER^ and subsequent increase in the calreticulin, CRT-1. Increased levels of CRT-1 result in aberrant accumulation of CA^2+^ ions in the ER. Interestingly, perturbations in *atf-6* result in decreased CRT-1, calcium efflux through the inositol triphosphate receptor, ITR-1, and increased mitochondrial CA^2+^ import, resulting in increased metabolic activity of mitochondria and extension of lifespan (Burkewitz et al., 2020).

### Impact of Mitochondrial Quality Control on the Heat Shock Response

Considering the close communication between the mitochondria and ER and the impact of the UPR^ER^ on mitochondrial health, it would be of great interest to determine the overlapping functions of all stress responses and how each impact the other. For example, induction of the UPR^MT^ has direct impact on the HSR. HSF-1 function declines during the aging process (Trivedi and Jurivich 2020) and the HSR can become dysfunctional as early as the second day of reproductive capacity in *C. elegans* (Labbadia and Morimoto 2015). However, one study found that low levels of mitochondrial stress through perturbation of a cytochrome C oxidase subunit, *cox-6c*, can increase functional capacity of the HSR at late age and has a positive impact on stress resilience and longevity. Specifically, activation of both the UPR^MT^ and HSR are both equally responsible for the increase in organismal health and lifespan found in animals exposed to mitochondrial stress (Labbadia et al., 2017). Interestingly, knockdown of *hsp-6* (the gene encoding the primary mitochondrial chaperone mtHSP70) also resulted in activation of the HSR. In fact, microarray analysis of animals with *hsp-6* knockdown found 187 genes differentially expressed, with 66 being known targets of HSF-1 and DVE-1, highlighting the distinct and important overlap between UPR^MT^ and HSR. Further investigation of these overlapping genes found that many genes were involved in lipid metabolism. Subsequent metabolomics identified that this mitochondria-to-cytosolic stress response (MCSR) was dependent on an overall increase in fatty acid levels. Specifically, increased cardiolipin and inhibition of ceramide synthesis were sufficient to drive MCSR induction. Most importantly, blocking fatty acid oxidation to increase fats using the drug, perhexiline, was sufficient to drive MCSR activation in both worms and human cells (Kim et al., 2016). This study has powerful translational potential, with perhexiline serving as a potential therapeutic intervention for aging and protein aggregation disorders, including Huntington’s, Alzheimer’s, and ALS. Finally, HSF-1 has been shown to directly impact mtDNA gene expression through elevated histone H4 levels. Specifically, knockdown of the conserved heat shock factor binding protein, *hsb-1* results in increased H4 levels early in development, which alters chromatin state of mtDNA, decreases expression of mtDNA-encoded genes, reduces mitochondrial respiratory capacity, and promotes lifespan in a UPR^MT^-dependent manner (Sural et al., 2020).

These studies continued to bridge the functional role of the mitochondria to other organelles, particularly with the surprising finding that HSF-1, the transcriptional regulator originally ascribed to HSR, now being directly influenced by UPR^MT^. HSF-1 has also been shown to directly impact the actin cytoskeleton (Baird et al., 2014; Higuchi-Sanabria et al., 2018b), and many studies have already highlighted the importance of actin as serving as the scaffold for–and regulating the dynamics of–the mitochondria (Fehrenbacher et al., 2004; Boldogh and Pon 2006; Higuchi et al., 2013; Korobova et al., 2013; Manor et al., 2015). A large-scale cross-organism screen combining the power of CRISPR-Cas9 screening in human cells (Shalem et al., 2014) and RNAi screening in *C. elegans* identified EPS-8 as a novel link between actin and mitochondrial homeostasis (Moehle et al., 2021). Specifically, knockdown of *eps-8* resulted in hyperstabilization of actin through the integrin, PAT-3. This increased stability of actin resulted in fragmentation of mitochondria and a beneficial activation of the UPR^MT^, such that these animals exhibited increased lifespan. These findings are conserved in human cells where hyperactive integrin signaling can hyperstabilize actin filaments, resulting in increased stress resilience through activation of UPR^MT^ through HSF1 and ATF5 (Tharp et al., 2021). These studies beg the question of what impact–if any–UPR^MT^ and mitochondrial dysfunction can have on the actin cytoskeleton. While one would expect that mitochondrial dysfunction and decreased energetics may negatively influence the actin cytoskeleton, the activation of the HSR and increased function of HSF-1 during mitochondrial stress may actually positively impact actin health and function.

### Overlapping Functions of Heat Shock Response and UPR^ER^

Similar to the overlapping functions of UPR^MT^ and HSR, the UPR^ER^ also shows some overlap. In fact, nine stress response genes are induced by both UPR^ER^ and HSR (Liu and Chang 2008), including the ER chaperone, HSP-4/BiP (Kohno et al., 1993) and the COPII cargo receptor, Erv29 (Caldwell et al., 2001). Importantly, the activation of the HSR via a constitutively active HSF1 rescued the growth defect of UPR^ER^ deficient cells. In addition, induction of the UPR^ER^ via heat-shock in mammalian cells can activate canonical XBP1 targets (Heldens et al., 2011). While these studies were not performed in *C. elegans*, overexpression of *xbp-1s* in *C. elegans* did seem to negatively impact the expression of key HSF-1 targets, including *hsp-16.2* and *hsp-70* (Taylor and Dillin 2013), clearly indicating that UPR^ER^ function has at least some indirect impact on HSR. More thorough analysis is certainly necessary to determine the direct ramifications of UPR^ER^ on HSR and vice versa during the aging process in *C. elegans*.

## Concluding Remarks

Overall, it is becoming increasingly clear that overlapping functions exist between stress responses and that each response can impact other organelles. For example, UPR^MT^ has direct ramifications on overall health and fitness of an organism–not just on the mitochondria, and not just on the single cell type exposed to mitochondrial stress. The same is true for the UPR^ER^ and the HSR, highlighting the critical importance of studying the cross-regulation of stress responses, and moving away from studying these quality control mechanisms in a black box of specificity. One intriguing question is to understand how all quality control machineries are regulated as a whole. For example, under conditions of competing needs, does there exist a preference for a specific stress response? While it is clear that the HSR is a critical response to thermal stress, heat can also damage the mitochondria and ER, so how does the cell prioritize HSR over UPR^ER^/UPR^MT^ activation? Under these more “generalized” stresses, how does the cell preferentially activate one response over the other? Even further, if multiple stress responses are activated, would this have a synergistic effect producing a hyper long-lived animal? Or would there be a detrimental effect when activating too many stress responses? Future studies must continue to focus on the direct and indirect effects of one stress response on all organelles and take into consideration the effects of organelle-to-organelle signaling and even cell-to-cell signaling in the cross communication and cross regulation of stress responses.
